# *MYB10* and *MYB72* Are Required for Growth under Iron-Limiting Conditions

**DOI:** 10.1371/journal.pgen.1003953

**Published:** 2013-11-21

**Authors:** Christine M. Palmer, Maria N. Hindt, Holger Schmidt, Stephan Clemens, Mary Lou Guerinot

**Affiliations:** 1 Department of Biological Sciences, Dartmouth College, Hanover, New Hampshire, United States of America; 2 Department of Plant Physiology, University of Bayreuth, Bayreuth, Germany; NC State University, United States of America

## Abstract

Iron is essential for photosynthesis and is often a limiting nutrient for plant productivity. Plants respond to conditions of iron deficiency by increasing transcript abundance of key genes involved in iron homeostasis, but only a few regulators of these genes have been identified. Using genome-wide expression analysis, we searched for transcription factors that are induced within 24 hours after transferring plants to iron-deficient growth conditions. Out of nearly 100 transcription factors shown to be up-regulated, we identified MYB10 and MYB72 as the most highly induced transcription factors. Here, we show that MYB10 and MYB72 are functionally redundant and are required for plant survival in alkaline soil where iron availability is greatly restricted. *myb10myb72* double mutants fail to induce transcript accumulation of the nicotianamine synthase gene *NAS4*. Both *myb10myb72* mutants and *nas4-1* mutants have reduced iron concentrations, chlorophyll levels, and shoot mass under iron-limiting conditions, indicating that these genes are essential for proper plant growth. The double *myb10myb72* mutant also showed nickel and zinc sensitivity, similar to the *nas4* mutant. Ectopic expression of *NAS4* rescues *myb10myb72* plants, suggesting that loss of *NAS4* is the primary defect in these plants and emphasizes the importance of nicotianamine, an iron chelator, in iron homeostasis. Overall, our results provide evidence that MYB10 and MYB72 act early in the iron-deficiency regulatory cascade to drive gene expression of *NAS4* and are essential for plant survival under iron deficiency.

## Author Summary

Iron deficiency is the most common human nutritional disorder, afflicting more than three billion people worldwide. Most of these people rely on plants for their dietary iron. Iron is also one of the three nutrients that most commonly limit plant growth. Despite progress in tracing how Fe moves throughout the plant, we still do not fully understand how plants sense and respond to Fe availability. Here, we report on two apparently redundant MYB transcription factors that are acting to control one gene that has a major impact on the ability of plants to grow in alkaline soil where iron availability is greatly restricted. In the absence of these transcription factors, plants are unable to survive on this soil. As 1/3 of the world's soils are alkaline, understanding how plants cope may enable the growth of crops on soils currently not suitable for agricultural production. These two transcription factors also extend the iron deficiency network and this work provides insight into the tight control plants exert over iron uptake and distribution.

## Introduction

Understanding plant growth and nutrient uptake is becoming increasingly critical in today's climate of rising food and energy demands. Iron is one of the most limiting nutrients for plant growth and is required for many essential processes including photosynthesis and respiration [1]. Although iron is abundant in most soils, it is often found in the bio-unavailable form of insoluble ferric oxides, particularly in aerobic or alkaline soils [2]. To overcome iron-limiting conditions, graminaceous plants utilize a chelation strategy in which iron-chelating compounds called phytosiderophores are released into the rhizosphere where they chelate iron. These complexes are then taken up via YS/YSL transporters [3], [4]. Non-graminaceous plants utilize a reduction strategy in which they acidify the soil by proton extrusion, reduce iron from the ferric to the ferrous form, and then take up the ferrous form via a ferrous iron transporter. In the model plant *Arabidopsis thaliana*, the H^+^-ATPase AHA2 has been shown to play a role in proton extrusion [5], the ferric chelate reductase FRO2 is responsible for reduction of ferric iron [6], and the high affinity ferrous iron transporter IRT1 takes up iron into the epidermis of the root [7], [8]. Once in the root, ferrous iron is thought to be loaded into the xylem by Ferroportin1 (FPT1) [9] where it forms a complex with citrate [10] and it is loaded into the phloem complexed to nicotianamine (NA) [11]. NA is synthesized from S-adenosyl methionine by nicotianamine synthase (NAS) and can form stable complexes with many metals including iron and nickel [12]. NA-Fe^2+^ complexes are essential for unloading of the vasculature as well as remobilizing iron [13], [14], highlighting these complexes as particularly essential for iron homeostasis. Recent work has also revealed an essential role of NA in zinc tolerance in Arabidopsis and in hyperaccumulation of zinc in *Arabidopsis halleri*, further stressing the emerging central role of NA in metal homeostasis and suggesting potential for this molecule in biofortification and bioremediation [15], [16]. Indeed, there are now a number of studies showing that expression of various *NAS* genes leads to increased levels of micronutrients in the seed [17]–[20]. For example, overexpression of the barley *hvNAS1* gene in rice leads to increased concentrations of iron and zinc in the grain [17]. Most encouragingly, enhancing the NA concentration does increase the levels of bioavailable iron and zinc in polished rice. [18].

Transcripts for many of the genes encoding iron homeostasis factors increase within 24 hours of exposure to iron deficiency [21], [22]. Up to 1000 genes show changes in transcript abundance in Arabidopsis roots in response to iron deficiency, but relatively little is known about the factors that may regulate them [21]–[23]. Previous work in Arabidopsis has shown that the bHLH transcription factor FIT (FER-like Iron-deficiency-induced Transcription factor) is required for mounting a proper iron-deficiency response, and *fit* plants are severely chlorotic, have reduced iron content, are seedling lethal under iron deficiency, and fail to induce approximately 75 genes that are normally induced under iron deficiency [23]–[25]. FIT has been shown to form heterodimers with four other bHLH transcription factors belonging to the group Ib sub-family (bHLH38/39/100/101), leading to activation of *FRO2* and *IRT1* [26], [27]. Another member of the bHLH transcription factor family, POPEYE (PYE), is induced in the root under iron deficiency and has been recently shown to be critical for proper iron homeostasis [28]. *pye*-1 mutants are able to survive under normal growth conditions, but the mutation is lethal when plants are grown under iron-deficient conditions. Unlike FIT, PYE appears to be a negative regulator and several iron-deficiency regulated genes show higher expression in *pye*-1 mutants.

To identify important transcriptional regulators, we searched for transcription factors that are induced within 24 hours of transferring plants to iron-deficient growth conditions. MYB10 and MYB72 are the most highly induced transcription factors within 24 hours of exposure to iron deficiency, and this induction continues to increase through 72 hours [22], [23]. Previous studies have shown that MYB10 and MYB72 are also induced in response to zinc and cadmium excess, conditions that are thought to induce iron deficiency [29]. MYB10 and MYB72 are close paralogs in the MYB family, a family with 126 members characterized by the R2 and R3 MYB helix-turn-helix DNA-binding domain [30]. In spite of the large size of this family in Arabidopsis, no MYB factors have been shown to play a functional role in iron homeostasis. In fact, no role has been found yet for MYB10. MYB72 has been shown to be required for induced systemic resistance (ISR) in Arabidopsis roots, although this may also be a result of iron stress, caused by resource competition between the plant and the pathogen [31], [32]. MYB10 and MYB72 are clustered together within the family tree and show 55% similarity overall with 87% similarity within the MYB domains [33]. Many closely clustered MYB family members show conserved function, which suggests that MYB10 and MYB72 may be functionally redundant [34]. At present, there is no information about the function of MYB10 or MYB72 orthologs in other plant species.

In this study, we establish that the transcription factors MYB10 and MYB72 are required for plant survival under iron deficiency. In the absence of these two MYB proteins, induction of the nicotianamine synthase gene *NAS4* is reduced in the root, leading to sensitivity to iron deficiency as well as to nickel or zinc excess. Our results establish an essential role for MYB10 and MYB72 in the regulatory cascade of the iron-deficiency response and highlight the importance of NAS4 for proper plant metal homeostasis. Given the emerging role of NA in metal homeostasis, these findings can offer insight into future application in biofortification or bioremediation.

## Results

### MYB10 and MYB72 are required for survival under iron deficiency

**Figure 1:**
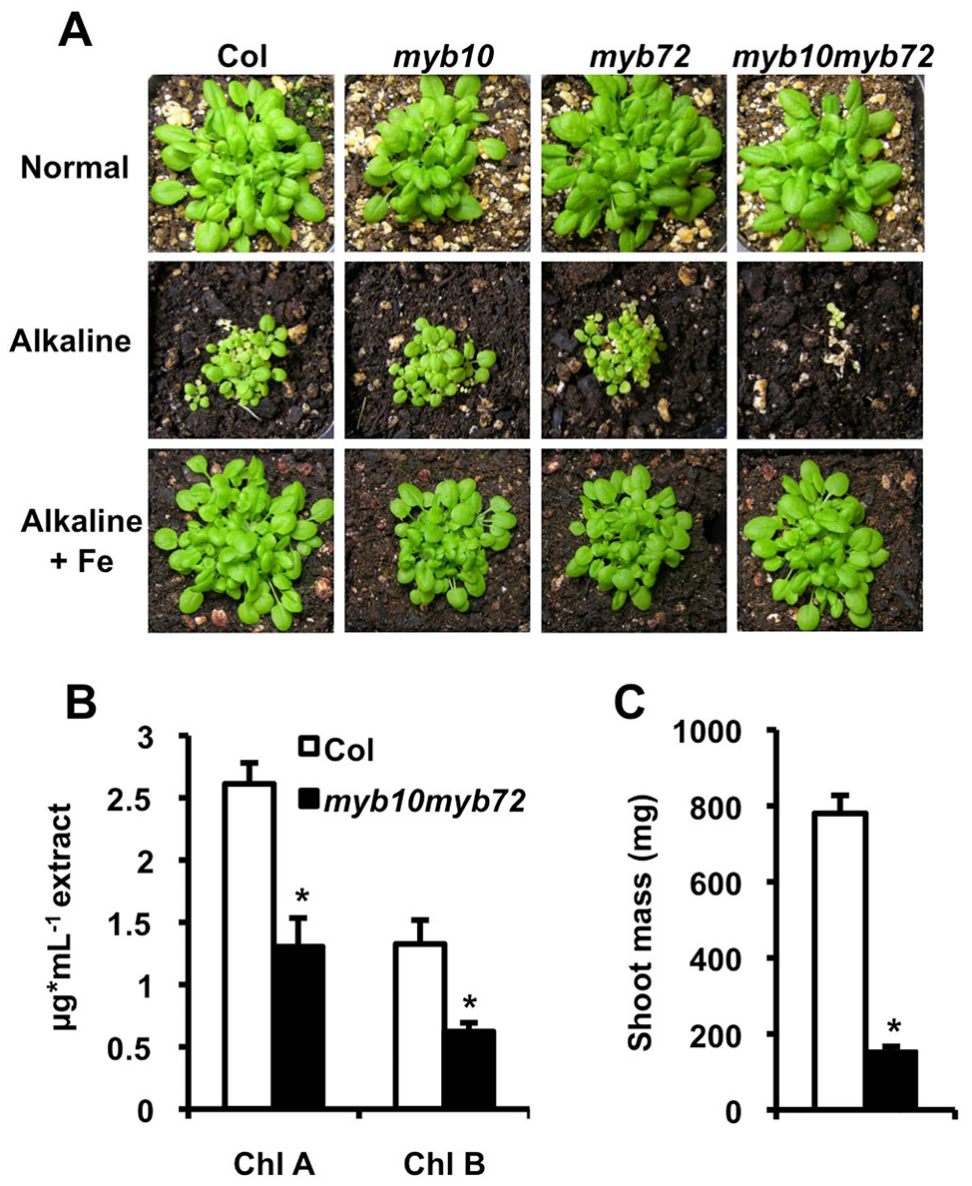
*MYB10* and *MYB72* are required under iron deficiency. **A.** Plants were grown for 3 weeks on soil under normal, alkaline (iron-limiting), and alkaline +Fe (iron excess) conditions. Iron was added by watering the plants with 500 µM FeEDDHA. **B.** Plants were grown on intermediate soil (½ normal, ½ alkaline) for 4 weeks and chlorophyll levels were measured. **C.** Plants grown as in **B.** Shoot mass of 15 pooled plants. n = 3 biological replicates. p values were calculated using Student's t-test to compare Col and *myb10myb72* plants. *p<0.01.

To determine if MYB10 and MYB72 function in the iron deficiency pathway, we obtained T-DNA insertion lines for *MYB10* and *MYB72* from the Salk collection [35] with an insertion 189 bp upstream of the start codon in the *myb10* allele or 296 bp downstream of the start of the third exon in the *myb72* allele (Figure S1A). These lines were crossed to create the double mutant *myb10myb72*. We confirmed that full-length transcripts of *MYB10* and *MYB72* were absent in these plants (Figure S1B). Although *myb10*, *myb72*, and *myb10 myb72* plants did not show visible mutant phenotypes when grown on normal soil (pH∼6.0), *myb10myb72* plants displayed seedling lethality when grown on alkaline soil (pH∼8.0), a growth condition which leads to iron deficiency due to decreased iron solubility at the elevated pH of the soil (Figure 1A) [2]. This lethality was not observed in *myb10* or *myb72* plants (Figure 1A), suggesting that MYB10 and MYB72 have overlapping roles. Seedling lethality of *myb10myb72* plants was rescued by supplementation with exogenous iron (Figure 1A), but not zinc or manganese, which are also limiting for plant growth under alkaline conditions (Figure S1C).

Iron is required for the synthesis of chlorophyll, and iron-deficient plants often show decreased levels of chlorophyll [36]. We measured chlorophyll levels in plants grown on intermediate pH soil (pH∼7.0) and found that while *myb10myb72* plants were able to survive on this soil, they showed significantly reduced chlorophyll levels (Figure 1B) and shoot mass (Figure 1C). Plants tightly regulate iron concentrations and while it is rare to see alterations in iron concentrations, concentrations of other metals such as zinc, manganese, cadmium, cobalt, and molybdenum can often serve as a fingerprint for iron deficiency [37]. We find that when grown in intermediate soil, *myb10myb72* plants have significantly reduced shoot iron and molybdenum concentrations, as well as significantly increased zinc, cadmium, and cobalt shoot concentrations (Table 1). Although a 13.5% decrease in shoot iron concentration relative to wild type may seem small, as mentioned above, plants tightly regulate their iron levels. The decrease in iron concentration in the *myb10myb72* mutant, coupled with the rescue of *myb10myb72* seedling lethality by the addition of iron to alkaline soil, suggests that low iron underlies the growth defect. *myb10myb72* mutants also have significantly lower iron concentrations and higher cadmium and zinc concentrations in the seeds when grown on normal soil, suggesting that in the absence of MYB10 and MYB72, the plant is unable to properly regulate iron levels (Table 1). No significant differences in iron concentration were detected in roots, but *myb10myb72* mutants show significantly higher concentrations of cadmium in their roots (Table S1). *myb10myb72* plants also show significantly reduced manganese concentrations in both normal and intermediate pH growth conditions, suggesting an additional role for these transcription factors in manganese homeostasis (Table S2). Excess zinc can also cause iron deficiency by competing for uptake [38]. *myb10myb72* plants were more sensitive to excess zinc levels, and this phenotype could be reversed by the presence of added iron (Figure S2).

**Table 1:** ICP-MS data for soil-grown plants.

	Fe	Cd	Co	Zn	Mo
**SHOOTS – normal soil**					
Col	110.5±3.6	0.48±0.08	0.46±0.10	156.6±5.3	1.22±0.07
*myb10myb72*	105.1±2.1	0.63±0.03	0.76±0.10	162.3±4.3	1.18±0.02
*nas4-1*	104.5±2.4	0.57±0.04	0.51±0.12	146.9±5.3	1.08±0.04
**SHOOTS - intermediate soil**					
Col	102.7±2.6	0.40±0.02	0.24±0.01	113.2±2.3	15.56±0.67
*myb10myb72*	88.9±4.4*	0.70±0.05*	0.65±0.11*	192.8±7.9*	8.85±0.56*
*nas4-1*	93.9±3.0*	0.51±0.03*	0.53±0.10*	119.8±2.3	12.74±0.90*
**SEEDS – normal soil**					
Col	39.8±0.94	0.04±0.00	0.010±0.002	48.8±1.0	1.32±0.04
*myb10myb72*	37.5±0.68*	0.10±0.01*	0.009±0.001	55.7±1.85*	1.44±0.03*
*nas4-1*	32.9±0.87*	0.10±0.01*	0.012±0.002	49.2±5.93	1.37±0.04

Metal levels in parts per million (ppm) were determined using ICP-MS analysis of tissue samples. Values represent mean ± SEM. n = 6 biological replicates.

* significantly different from Col (p≤0.05).

### *MYB10* and *MYB72* are expressed in the root under iron deficiency

**Figure 2:**
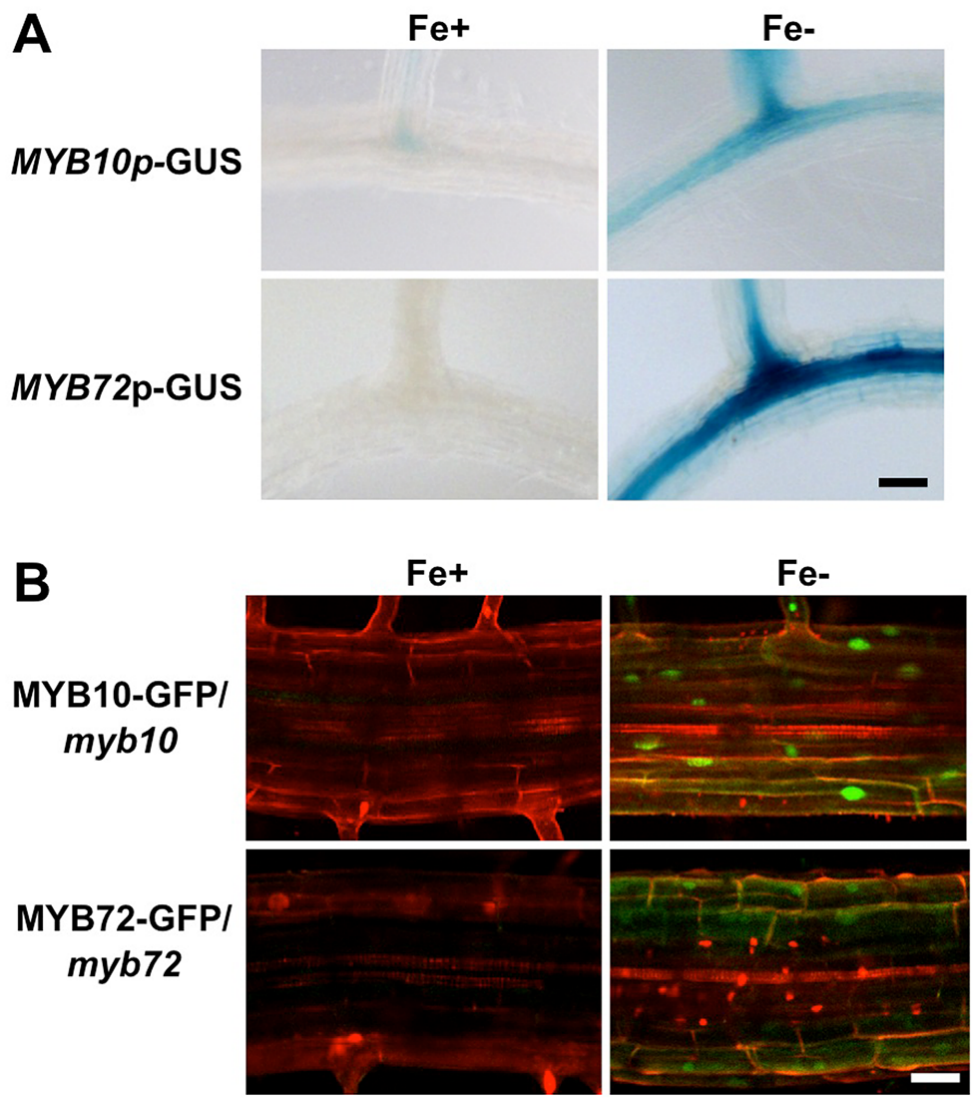
*MYB10* and *MYB72* are expressed in the root under iron deficiency. **A.** GUS expression in the roots of stable *MYB10*-GUS and *MYB72*-GUS lines in the Col background. Plants were grown for 5 d on ½ B5 and transferred to +/− Fe plates for 72 hrs. **B.** GFP expression in the roots of *myb10* or *myb72* plants stably transformed with MYB10-GFP or MYB72-GFP protein fusions, respectively. Plants were grown for 5 d on ½ B5 and transferred to +/− Fe plates for 72 hrs. Roots were counterstained with propidium iodide (red). **A. B.** Differentiation zone of root. Scale bar is 0.5 mm.

Previous transcriptional profiling in roots of plants exposed to iron deficiency showed that steady state levels of *MYB10* and *MYB72* transcripts are increased in whole roots under iron deficiency, but showed no enrichment when analyzed by tissue layer [22], [23]. To determine where *MYB10* and *MYB72* are expressed at the tissue level, we generated transgenic plants expressing the ß-glucuronidase (GUS) reporter gene fused to the *MYB10* or *MYB72* endogenous promoters (*MYB10*-GUS or *MYB72*-GUS). We show that *MYB10*-GUS and *MYB72*-GUS were strongly expressed in the root stele when plants were exposed to iron-deficient conditions for 72 hours (Figure 2A). *MYB10*-GUS also shows staining in the outer layers of the lateral roots under iron deficiency. No staining was observed in other tissues, consistent with expression datasets from AtGenExpress and Genevestigator [39], [40]. To localize the MYB10 and MYB72 proteins, we fused GFP to the C terminus of each coding region with expression driven by the endogenous promoter. These constructs were stably transformed into *myb10* or *myb72* mutant plants, respectively, and were able to complement the mutant phenotype of *myb10myb72* plants, indicating that the constructs are functional (Figure S1D). Consistent with GUS staining patterns, no signal was detected in roots of plants grown under iron sufficiency. MYB10-GFP and MYB72-GFP were detected in nuclei of root cells under iron deficiency with some staining also seen in the cytoplasm and signal was detected in multiple cell layers of the root (Figure 2B).

### *NAS4* is misregulated in *myb10myb72* plants

**Figure 3:**
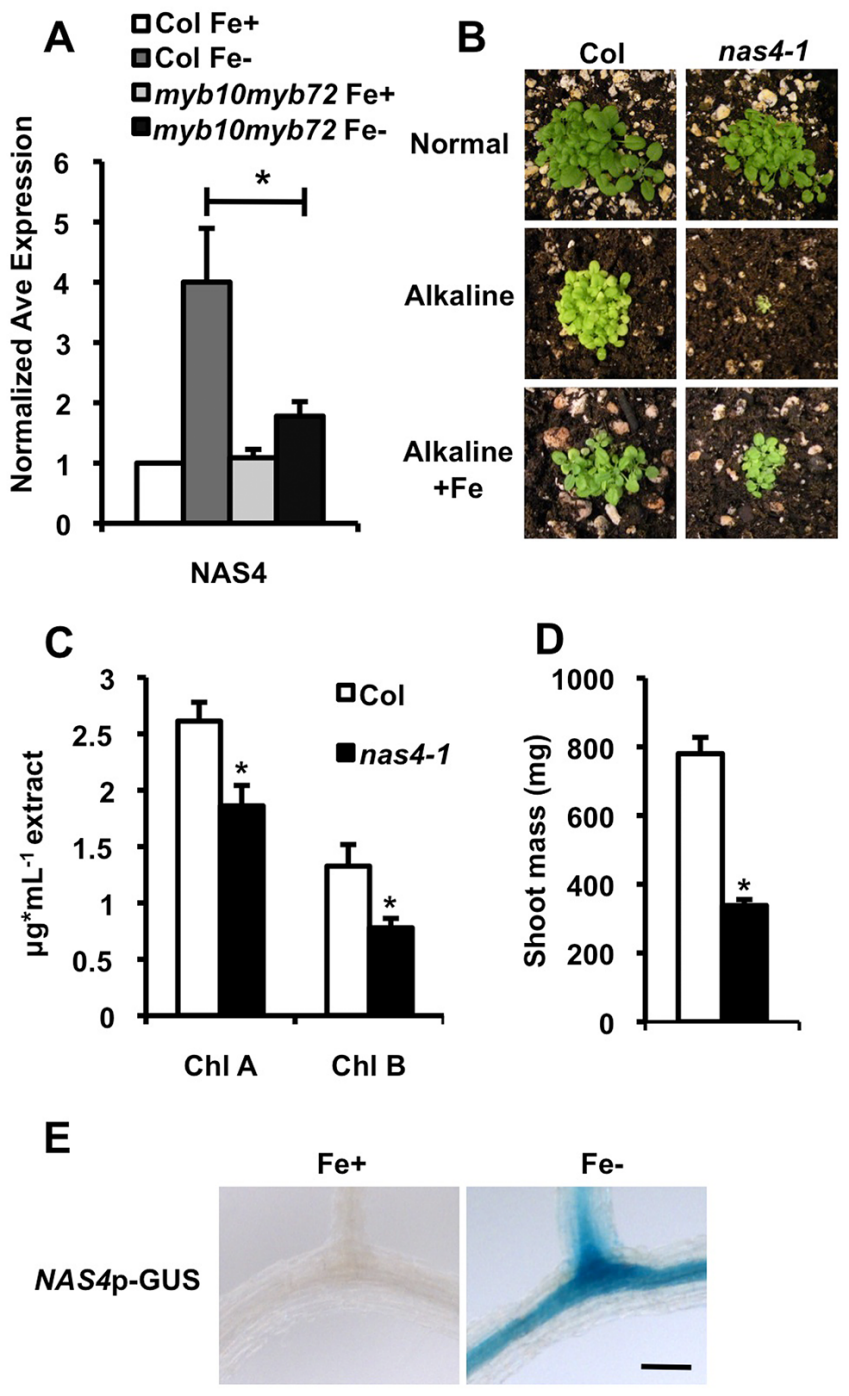
*nas4-1* mutants are sensitive to alkaline soil. **A.** qPCR on root tissue from plants grown for 2 weeks on ½ B5 and transferred to +/− Fe plates for 72 hrs. **B.** Plant seedling phenotype on soil under normal, alkaline (iron-limiting), and alkaline +Fe (excess iron) conditions. **C.** Chlorophyll levels of 15 pooled plants grown on intermediate soil for 4 weeks. **D.** Quantification of shoot mass of 15 pooled plants grown on intermediate soil for 4 weeks. **E.** GUS expression in the roots of stable *NAS4p*-GUS lines in the Col background. Scale bar is 0.5 mm. Plants were grown for 5 days on ½ B5 and transferred to −Fe plates for 72 hr. n = 3 biological replicates. *p<0.01.

To identify targets of the MYB10 and MYB72 transcription factors, we conducted microarray analysis on root tissue after 2 week-old seedlings were exposed to iron deficiency for 72 hours. In contrast to the extensive transcriptional misregulation often seen with the loss of a transcription factor, loss of MYB10 and MYB72 in iron-deficient roots led to very few expression changes. 9 genes were found to have at least a 2-fold difference in expression level when comparing RNA isolated from wild type and *myb10myb72* root tissue from plants grown under iron deficiency (Table S3). Of these genes, only 5 were confirmed by qPCR to be misregulated in *myb10myb72* plants (Figure 3A, Figure S1B, S3A). The two most misregulated transcripts were *MYB10* and *MYB72*, confirming the lack of transcript in *myb10myb72* mutants. The transcript levels of the 3 remaining genes, *At5g38910*, *At4g33666*, and *NAS4*, were also significantly lower in *myb10myb72* tissue, suggesting that MYB10 and MYB72 positively regulate their transcription under iron deficiency. While this is a surprisingly small number of targets, all three have potential roles in metal homeostasis. *At5g38910* is predicted to a be a putative Mn superoxide dismutase, *At4g33666* is an unknown protein predicted to localize to the chloroplast, and *At1g56430* encodes NAS4, a synthase of NA, a well-known chelator of iron. *At5g38910* is not iron-regulated, so we did not pursue this gene in our studies, but both *At4g33666* and *NAS4* normally show increased transcript levels in the root under iron deficiency [22], [23]. To further assess whether these genes might be direct targets, we analyzed the promoter regions for consensus MYB binding sites. MYB10 and MYB72 belong to group C of the MYB family and are predicted to bind to the IIG canonical sequence of 5′-G(G/T)T(A/T)GGT(A/G)-3′ [41]. Of the three confirmed targets, only *NAS4* has a binding site within 1 kb upstream of the predicted translational start site.

In addition to microarray analysis, we used qPCR to confirm that MYB10 and MYB72 are not required for expression of *FRO2* and *IRT1*, the two main genes required for mounting a proper iron deficiency response. Both *FRO2* and *IRT1* were induced in *myb10 myb72* plants under iron deficiency. In fact, levels of ferric chelate reductase activity and IRT1 protein were somewhat elevated under iron deficiency in the *myb10/myb72* plants, suggesting that *myb10/myb72* plants are iron deprived relative to wild type plants. We also investigated if MYB10 and MYB72 regulate the other two root-expressed *NAS* genes, *NAS1* and *NAS2*, and found that while *NAS2* is modestly induced in wild type plants under iron deficiency, this induction is completely lost in the *myb10myb72* mutants (Fig. S3B). *NAS1* expression was not altered in the double mutant background. *NAS2* also has a MYB binding site within 1 kb upstream of the translational start site. These data suggest that in addition to *NAS4*, *NAS2* is also a downstream target of MYB10 and MYB72 under iron deficiency.

### *nas4-1* mutants are sensitive to iron deficiency

To test if expression of *NAS4*, *NAS2*, or *At4g33666* is required for survival under iron deficiency, we obtained T-DNA insertional mutants from the SALK collection [35]. All three genes are encoded by a single exon, and the T-DNA insertion is found in the exon of each mutant line. qPCR showed dramatically reduced transcript levels of the interrupted gene in the *a4tg33666*, *nas4-1*, and *nas2-2* lines (Figure S3C, D) [42]. *at4g33666* mutants showed no visible phenotype on alkaline soil, suggesting that *At4g33666* is not required for survival under iron deficiency (Figure S4A). We found no detectable phenotype for the *nas2-2* mutants when grown on alkaline soil (Figure S4B). However, we find that *nas4-1* mutants are seedling lethal on alkaline soil, and this lethality can be rescued by application of exogenous iron (Figure 3B), similar to the phenotype seen in *myb10myb72* plants. *nas4-1* mutants survive on intermediate soil, but show reduced leaf size and interveinal chlorosis (Figure 3C, D). This chlorosis is limited to the interveinal region (Figure S4C). Like *myb10myb72* plants, *nas4-1* plants also have significantly reduced shoot iron and molybdenum concentrations and increased shoot cadmium and cobalt concentrations when grown on intermediate soil, as well as reduced seed iron concentrations and increased seed cadmium concentrations when grown on normal soil (Table 1). *nas4-1* plants also show decreased manganese concentrations (Table S2) and sensitivity to excess levels of zinc (Figure S2). Lastly, we fused the *NAS4* promoter to the GUS reporter gene and see that *NAS4* is strongly expressed in the root stele under iron deficiency in the same pattern as *MYB10* and *MYB72* (Figure 3E). These data suggest that *NAS4* expression is required for proper iron homeostasis under iron-deficient growth conditions and that reduced expression of *NAS4* in *myb10myb72* plants may be the primary cause of the *myb10myb72* mutant phenotype.

### *myb10myb72* and *nas4-1* mutants are sensitive to nickel

**Figure 4:**
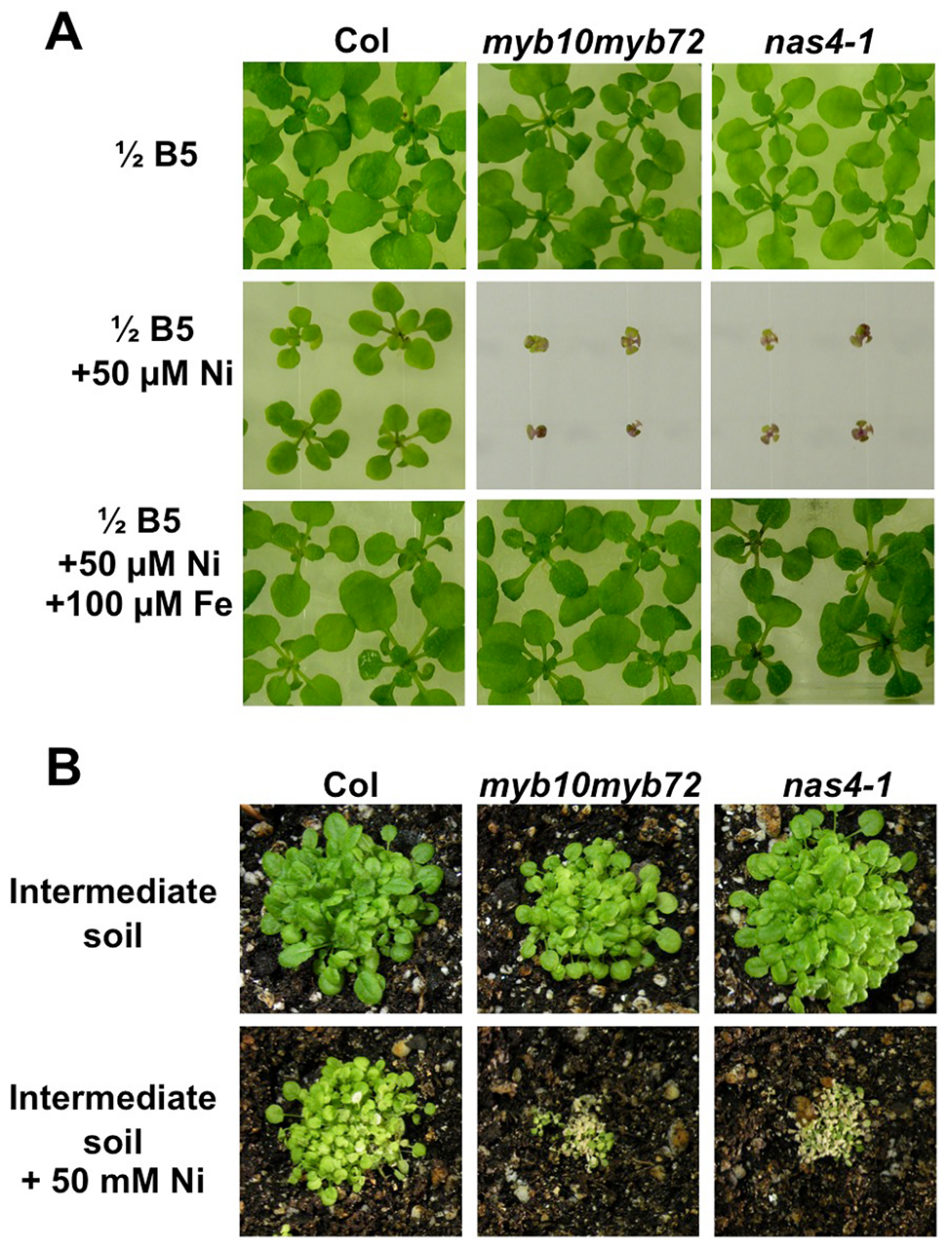
*myb10myb72* and *nas4-1* mutants are sensitive to excess nickel. **A.** Plants were grown for 3 weeks on ½ B5 plates supplemented as indicated. **B.** Plants were grown on intermediate soil for 3 weeks supplemented weekly with water or 50 mM NiSO_4_.

*NAS* genes have previously been implicated in nickel tolerance [43]–[45], so we tested the sensitivity of *nas4-1* and *myb10myb72* plants to excess levels of nickel. We found that *nas4-1* and *myb10myb72* plants showed severe growth defects in comparison to wild type when germinated on plates with excess nickel (Fig. 4A). This could be rescued by the addition of extra iron. Neither *nas2-2* nor *at4g33666* plants showed sensitivity (Figure S4D, E). *nas4-1* and *myb10myb72* plants also showed greater sensitivity than wild type plants to acute application of excess nickel when grown on soil (Fig. 4B).

### Overexpression of *NAS4* rescues *myb10myb72* mutants

**Figure 5:**
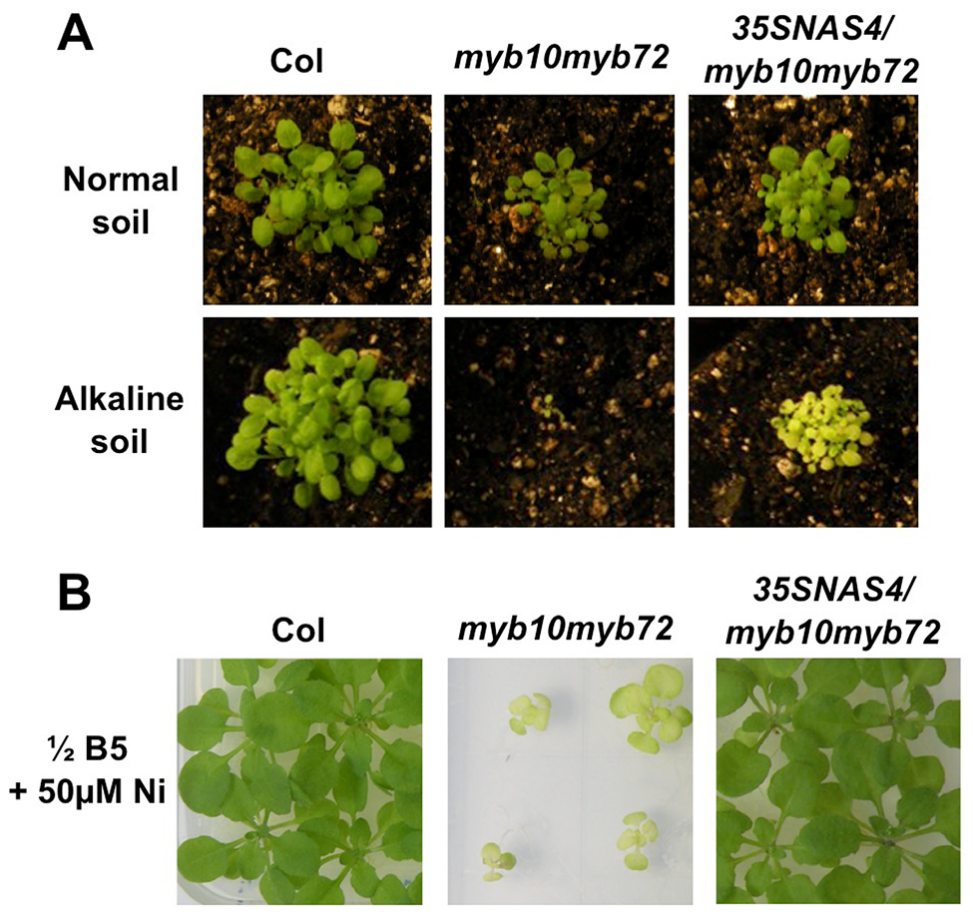
Overexpression of *NAS4* rescues *myb10myb72* sensitivity. **A.** Plants were grown for 3 weeks on soil under normal or alkaline (iron-limiting) conditions. **B.** Plants were grown for 3 weeks on ½ B5 plates with 50 µM NiCl_2_.

If the inability to induce *NAS4* expression is the primary cause of the iron and nickel sensitivity of *myb10myb72* plants, overexpression of *NAS4* should rescue *myb10myb72* plants. Because the endogenous *NAS4* promoter may require MYB10 and MYB72 for activation, we generated *myb10myb72* plants expressing *NAS4* under the control of the 35S promoter. These seedlings have high levels of *NAS4* expression and contain 4 fold more NA than wild type or *myb10myb72* seedlings (Figure S5). They survived on alkaline soil and showed rescue of nickel sensitivity (Figure 5A, B). It is important to note that under the growth conditions used here, both wild type and the *myb10 myb72* mutant have low levels of *NAS4* expression and hence low NA levels. It is the inability of *myb10 myb72* to raise its NA levels under Fe deficiency that is apparently causing a problem as increasing NA by overexpressing NAS4 clearly improved growth. Of course, because we do not see complete rescue of the *myb10myb72* mutant by *NAS4* it is possible these two transcription factors control other aspects of growth in alkaline soil. It is also possible that NA levels are not necessarily at the right concentration in the right place at the right time.

To test if MYB10 and MYB72 act directly on *NAS4*, we created a yeast line with stable integration of 1.5 kb of the NAS4 promoter driving the histidine biosynthesis gene *HIS3* and separately driving ß-galactosidase (LacZ). These stable lines were transformed with constructs expressing MYB10 or MYB72 fused the yeast GAL activation domain (AD). Yeast expressing MYB10-AD or MYB72-AD showed an increased ability to grow in the absence of HIS in the presence of the histidine competitor 3-Amino-1,2,3-triazole (3-AT), indicating that they bind to the *NAS4* promoter and drive HIS production (Figure S6A, B). This binding may be weak as there was no detectable activation of the LacZ reporter in these strains (Fig. S5B). We also carried out ChIP-qPCR and see enrichment of MYB10-GFP at the *NAS2* and *NAS4* promoters (Figure S6C).

## Discussion

**Figure 6:**
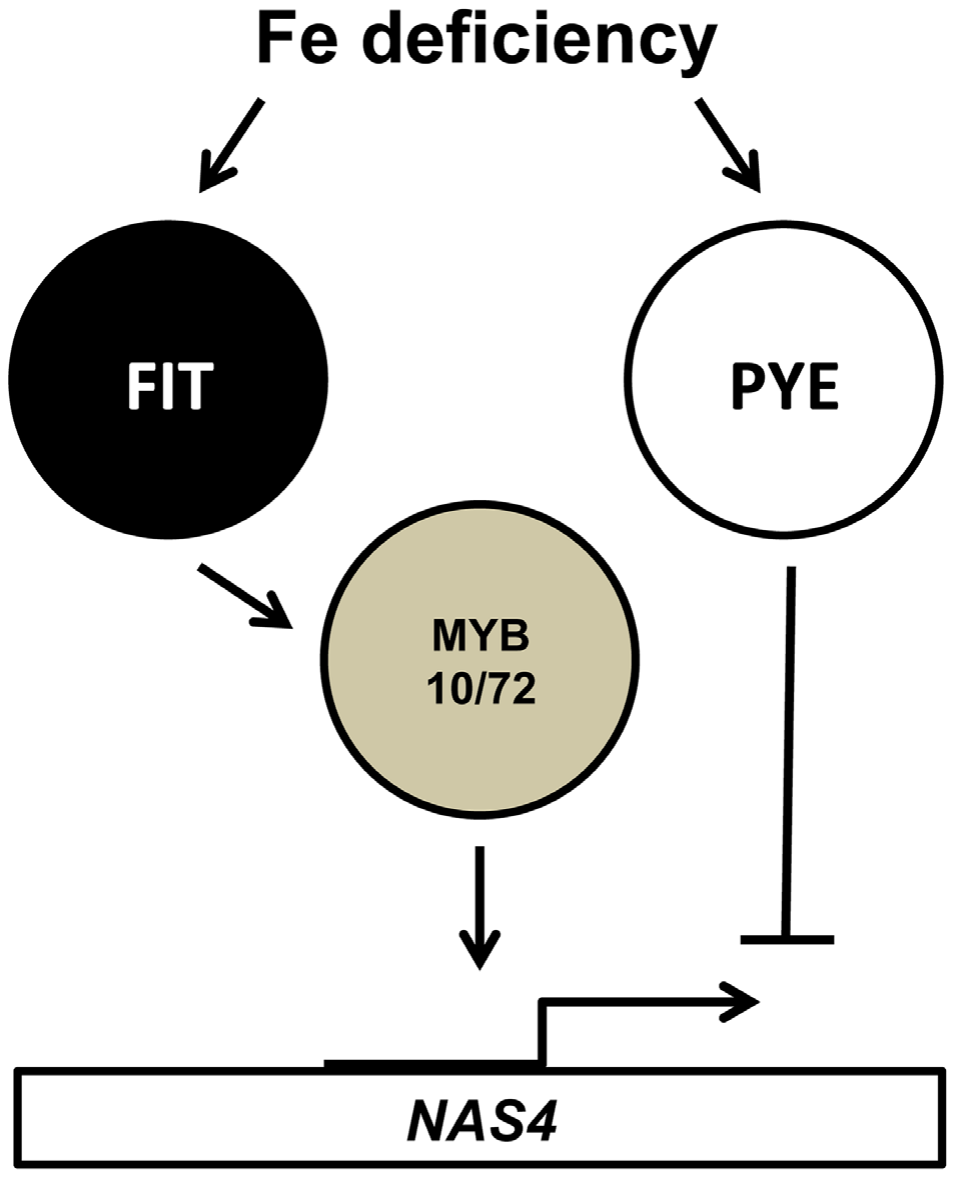
Relationship of MYB10, MYB72, FIT and PYE in the transcriptional response to iron deficiency. All interactions shown are believed to be direct binding to the respective promoters.

To date, a number of bHLH transcription factors, including FIT and PYE, have been shown to have a critical role in the iron-deficiency response in Arabidopsis [23], [27], [28]. Here we identify two new MYB family transcription factors, MYB10 and MYB72, that are required for plant survival under iron-limited conditions. *MYB10* and *MYB72* are both induced in the root within 6 hours of exposure to iron deficiency and this induction is reduced in *fit* mutants but not in *pye* mutants, suggesting that they act early in the regulatory cascade ([22], [23]; see model in Figure 6). MYB72 has recently been shown to be a direct target of FIT [46]. FIT is only expressed in the root and we see no expression of MYB10 or MYB72 in the shoot. We show that MYB10 and MYB72 are required for proper induction of *NAS4* expression in the root under iron deficiency. *NAS4* has previously been shown to be negatively regulated by PYE, suggesting that PYE may play an antagonistic role to that of MYB10 and MYB72 [28]. Loss of NA has previously been shown to lead to severe chlorosis in tomato *chloronerva* mutants which lack any functional NAS, as well as in tobacco plants overexpressing the nicotianamine amino transferase gene *NAAT*, which consumes NA [11], [47], [48]. Loss of all four *NAS* genes in *Arabidopsis thaliana* has also been shown to lead to chlorosis and sterility [42]. While previous work had shown that loss of any single *NAS* gene in Arabidopsis did not lead to a detectable phenotype under iron deficiency [42], [49], a recent review article reports that all single *nas* mutants are more chlorotic than wild type [12]. We find that *nas4-1* mutants show interveinal chlorosis and reduced shoot mass under iron-deficient conditions, as well as decreased shoot and seed iron concentrations. We confirmed previous results showing that *nas4-1* mutants are sensitive to high nickel levels (Figure 4) [42]. Overexpression of a *Thlaspi caerulescens NAS* or barley *NAS* in Arabidopsis has been shown to confer tolerance to excess levels of nickel [43], [44] and the nickel hyperaccumulator *Thlaspi caerulescens* has higher levels of *NAS4* expression than Arabidopsis [29], [45]. Most recently, Koen et al. [50] have also documented a role for *nas4* in the iron deficiency response as well as in sensitivity to cadmium.

Although previous work showed no reduction in total NA levels in *nas4-1* mutants [42], Koen et al. have reported that the concentration of NA in roots and shoots of *nas4* plants are significantly reduced compared to wild type [50]. Of the four *NAS* genes, *NAS4* is the most highly induced in the root under iron deficiency and is the only one that is induced in the stele, supportive of a role in iron transport with the root vasculature [22]. Nicotianamine has been well established to bind the divalent cations Mn<Fe<Zn<Ni, with increasing binding affinity [11]. Under conditions of limited iron, the limiting levels of NA will favor the formation of NA-Zn^2+^ complexes over Fe and Mn, leading to decreased iron and manganese transport into the phloem by the appropriate transporter. Excess nickel would exacerbate this decreased iron transport by competing for binding with the NA that is present, especially given the higher binding affinity.

We propose that the sensitivity of *myb10myb72* mutants to iron deficiency and excess nickel is predominantly due to reduced levels of *NAS4* and a resulting decrease in root vasculature levels of NA. This reduction is further exacerbated by reduced levels of *NAS2* in the root under iron deficiency. *NAS2* has been shown to be primarily expressed in the root epidermis and cortex [22]. We hypothesize that it provides a pool of NA in the epidermis where metals are taken up from the rhizosphere. Loss of *NAS2* alone does not lead to a detectable defect, but when coupled with loss of *NAS4*, root levels of NA are insufficiently induced under iron deficiency to meet the demands of the plant. The reduced pool of local NA also leads to an iron-deprived phenotype in these mutants under conditions of nickel excess due to competition for NA binding. Differences between the *myb10myb72* mutant and *nas4-1* may be attributable to the fact that *myb10myb72* still has *NAS4* expression in the shoot, which would allow for unloading of metals from the vasculature, leading to the uniform chlorosis seen in the *myb10myb72* mutants.

We put forward the following model. Under conditions of iron deficiency, upregulation of IRT1 leads to increased uptake of iron, zinc, cadmium, and manganese in the root epidermis. Local NA, potentially produced by NAS2, can bind these metals and either serves as a substrate for transporters that drive sequestration of excess metals into the vacuole or can move through the symplastic space toward the pericycle. Once in the pericycle, any unbound metals can be chelated by a second pool of NA, likely produced by NAS4, and loaded into the vasculature. Excess zinc and cadmium can be loaded into the xylem by HMA transporters [51], independent of NA levels. Once in the vasculature, NA complexes are taken into the shoot via YSL transporters and ultimately reach the seed, and this transport is dependent on shoot expression of *NAS4*. In the absence of MYB10 and MYB72, *NAS2* and *NAS4* are not properly induced under iron deficiency, leading to local decreases in NA, decreased levels of NA bound Fe^2+^, and ultimately decreased levels of iron in the shoot and the seed. Thus, we propose that MYB10 and MYB72 serve essential roles in the iron-deficiency response by regulating expression of *NAS2* and *NAS4* in the root. In combination with previous research, these findings suggest that the iron-deficiency induced transcription factors may induce distinct portions of the iron-deficiency response, from upregulation of the iron transporter as with FIT and IRT1 to up-regulation of the NA biosynthesis enzymes as with MYB10, MYB72 and NAS4. This partitioning by a small number of initial response transcription factors can allow the plant to fine-tune responses to the environment while still maintaining a rapid response.

In summary, our study shows that MYB10 and MYB72 are required for survival under iron-limiting conditions and nickel excess, for expression of *NAS2* and *NAS4*, and for proper iron acquisition in plants.

## Materials and Methods

### Plant materials and growth conditions

Seeds were surface sterilized and stratified for 2 d in the dark at 4°C. ½ B5 medium was supplemented with 1 mM MES, 2% sucrose, 0.6% agar, pH 5.8. To test for metal sensitivity, ½ B5 medium was amended with either 25 µM ZnSO_4_, 2.5 µM CdSO_4_ or 50 µM NiCl_2_. +Fe and −Fe minimal medium plates were made as described [52], 0.6% agar, and 1 mM MES, pH 6.0, then supplemented with either 50 µM Fe(III)-EDTA (+Fe), or 300 µM ferrozine [3- (2-pyridyl)-5,6-diphenyl-1,2,4-triazine sulfonate] (−Fe). Alkaline soil was prepared by addition of calcium oxide to a final soil pH of 7.5–8.0. “Alkaline +Fe” conditions were achieved by watering plants growing in alkaline soil with 500 µM FeEDDHA. Plants were grown under a 16/8-h light-dark cycle at 21°C.

### Identification of mutant lines

*myb10* and *myb72* lines were acquired from the Arabidopsis Knockout Facility and screened using *MYB10-* and *MYB72*-specific primers and a T-DNA–specific primer (Table S4) [53]. The Col lines *myb10* (SALK_120297), *myb72* (SALK_052993), *nas1-2* (SALK_082176), *nas2-2* (SALK_066962), *nas3-2* (SAIL_626_G10), *nas4-1* (SALK_130557) *nas4-2* (SALK_017016) were acquired from the Salk Collection and *at4g33666-1* (CS900982) from the WiscDsLoxHs Collection via ABRC [35], [54].

### GUS histochemical staining

*MYB10*-GUS and *MYB72*-GUS plants were grown for 5 d on ½ B5 medium and then transferred to +Fe or −Fe minimal media for 3 d. Plants were incubated with the substrate 5-bromo-4-chloro-3-indolyl β-D-glucuronide as described [55].

### GFP localization

MYB10-GFP and MYB72-GFP plants were grown for 5 d on ½ B5 medium and transferred to +Fe or −Fe minimal media for 3 d. Roots were counterstained with propidium iodide and visualized using an epifluorescence microscope, Nikon Eclipse 80i (Nikon USA), using 543 nm and 633 nm HeNe lasers with filter sets 31001 (exciter, D480/20x; dichroic, 505DCLP; emitter, D535/40m) and 31003 (exciter, D546/10x; dichroic, 560DCLP; emitter, D590/30m) from Chroma Technology.

### Chlorophyll assay

Plants were grown under a 16/8-h light-dark cycle at 21°C on ½ B5+2% sucrose for 2 weeks and then transferred to +Fe or −Fe minimal media for 3 d. The shoots were harvested and assayed for chlorophyll and carotenoid content as previously described [56].

### Real-time quantitative PCR

RNA was prepared from Col and *myb10myb72* plants (grown on ½ B5 for 2 weeks and then transferred to +Fe or −Fe minimal media for 3 d). Real-time quantitative PCR was performed on an ABI Model 7700 using SYBR Premix ExTaq (Perfect Real Time) reagents and protocol (Takara). Primers used are listed in Table S4. Samples were run in triplicate, normalized to *EF1α*, and arbitrary transcriptional units calculated. Values represent the average of three biological replicates.

### Microarray analysis

Plants were grown for 2 wk on ½ B5+2% sucrose and then transferred to −Fe plates for 72 hr under a 16/8-h light-dark cycle at 21°C. RNA was harvested from root tissue from 20 plants per replicate, and samples were run in duplicate. RNA was labeled and hybridized to the GeneChip Affymetrix Arabidopsis ATH1 Genome Arrays at Purdue University. Raw data were analyzed using the limma and affy packages in R [57]. Data has been deposited at ArrayExpress.

### Tissue elemental analysis

Tissue samples were dried at 92°C for 20 h in Pyrex tubes (16×100 mm). Elemental analysis was done by ICP-MS at Purdue University as described [58].

### Plasmid construction and plant transformation

For GUS constructs, *MYB10*, *MYB72*, and *NAS4* promoter regions were amplified from Col genomic DNA and cloned into the pMDC162 Gateway destination vector [59] using Gateway LR Clonase II (Invitrogen). *Agrobacterium tumefaciens* strain GV3101 was transformed with these constructs and used to transform plants using the floral dip method [60]. Transformants were isolated by selection on hygromycin (25 mg/mL).

35S lines were created by amplifying the *MYB10*, *MYB72*, and *NAS4* CDS off cDNA from wild-type roots grown for 2 weeks on half-strength B5 and then transferred for 3 d to −Fe minimal media. They were subcloned into the pENTR Gateway plasmid (Invitrogen) and sequenced. The *MYB10*, *MYB72*, *NAS4* constructs were subcloned into the pMDC32 Gateway destination vector [59] using Gateway LR Clonase II (Invitrogen). Proper integration was assessed by sequencing. Plant transformants were generated as above.

*MYB10-GFP* and *MYB72-GFP* constructs were made by subcloning the region starting from 1.5 kb upstream of the start site (promoter) and ending one codon upstream of the stop site in the open reading frame into the pENTR Gateway plasmid (Invitrogen), allowing for a translational fusion with GFP in the final vector. These constructs were then subcloned into a modified pEARLEY301 [61] that had the HA tag replaced with the GFP from pEARLEY103 (Suna Kim) using Gateway LR Clonase II (Invitrogen). Proper integration was assessed by sequencing. Constructs were amplified and prepared for direct use in protoplasts using a plasmid midi kit (Promega).

### Yeast one-hybrid

1.5 kb of the *NAS4* promoter was cloned into the pMW2 and pMW3 backbone and stably transformed into the YM4271 yeast strain. The *MYB10* or *MYB72* CDS was cloned into pGADT7 and transiently expressed in the stable transformants. Growth on selective media was assessed as described [62].

### Chromatin immunoprecipitation assay

Plants were germinated on ½ B5 plates with hygromycin (25 mg/mL) and no sucrose. After 14 d, they were transferred to −Fe minimal medium for 3 d. Roots from 40 plants were pooled and processed for chromatin immunoprecipitation as previously described [63]. Briefly, roots were cross-linked using formaldehyde, chromatin was extracted and sonicated to approximately 0.5–1 kb DNA fragments. Proteins were immunopreciptated using anti-GFP antibody (ab290, Abcam) and protein A agarose with salmon sperm (Millipore). Samples were washed, reverse crosslinked, precipitated, and analyzed for bound DNA using qPCR.

### Nicotianamine measurement

Plants were grown on 1/2 B5 plates for 2 weeks. Seedlings were harvested and 100 mg fresh weight was flash frozen and lyophilized overnight (n = 4 per genotype). Nicotianamine was quantified following derivatization with 9-fluorenylmethyl N-succinimidyl carbonate (Fmoc-Osu) by stable isotope dilution as previously described [64].

## Supporting Information

Figure S1 *MYB10* and *MYB72* are required under iron deficiency. A. Model of T-DNA insertion lines. Positions indicate distance from start codon. B. Relative transcript levels (RTL) as determined by qPCR on root tissue from plants grown for 2 weeks on ½ B5 and transferred to +/− Fe plates for 72 hrs. C. Plants were grown on alkaline soil (pH∼8) for 3 weeks and watered once a week with water, 500 µM FeEDDHA, 500 µM MnSO_4_, or 100 µM ZnSO_4_. D. Plants were grown for 3 weeks on ½ B5 plates with 50 µM NiCl_2_. *p<0.01.
(TIF)

Figure S2 *myb10myb72* and *nas4-1* mutants are sensitive to excess zinc. A. Plants were grown for 3 weeks on ½ B5 plates supplemented as indicated above. B. Plants were grown on normal soil for 4 weeks and then soaked daily with 35 mM ZnSO_4_ for 11 days.
(TIF)

Figure S3 Identifying targets of MYB10 and MYB72. A., B. qPCR on root tissue from plants grown on ½ B5 for 2 weeks and transferred to +/−Fe for 72 hr. *NAS3* is not expressed in the root. *p<0.05 C., D. qPCR analysis on roots of plants grown on ½ B5 for two weeks.
(TIF)

Figure S4 Analysis of other targets and *NAS* genes. A. Plants were grown on normal or alkaline soil for three weeks. B. Plants were grown for three weeks on alkaline soil. Homozygous mutants were tested from *nas1-2* (SALK_082176), *nas2-2* (SALK_066962), *nas3-2* (SAIL_626_G10), and *nas4-1* (SALK_130557). C. The fifth rosette leaf of plants grown as in B. D., E. Plants were grown on ½ B5 for 3 weeks and supplemented with 50 µM NiCl_2_ as indicated.
(TIF)

Figure S5 Overexpression of *NAS4* increases NA content in *myb10 myb72* plants. A. qPCR on plants grown for 2 weeks on 1/2 B5. B. NA content of same plants used in panel A.
(TIF)

Figure S6 *NAS2* and *NAS4* are likely direct targets of *MYB10/MYB72*. A. Yeast stably expressing *NAS4p-HIS3*, *NAS4p-LacZ* were transformed with *Gal-AD*, *MYB10AD*, and *MYB72-AD* constructs. DNA was extracted and construct presence was confirmed by PCR. B. Colonies were grown on triple dropout selection plates and replica-plated onto media containing increasing concentrations of the HIS3 competitor 3AT and onto filter paper incubated with the β-gal substrate. C. ChIP analysis of the *NAS2* and *NAS4* promoter regions.
(TIF)

Table S1 ICP-MS data for hydroponically-grown roots.
Metal levels in parts per million (ppm) were determined using ICP-MS analysis of tissue. Values represent mean ± SEM. n = 4 biological replicates. *significantly different from Col (p≤0.05).
(DOCX)

Table S2 ICP-MS data for soil-grown plants. Metal levels in parts per million (ppm) were determined using ICP-MS analysis of tissue. Values represent mean ± SEM. n = 4 biological replicates. *significantly different from Col (p≤0.05).
(DOCX)

Table S3 1.5-fold misregulated genes by microarray. Plants were grown for 2 wk on ½ B5+2% sucrose and transferred to −Fe conditions for 72 hr. RNA was prepared from root tissue and hybridized to GeneChip Affymetrix Arabidopsis ATH1 Genome Arrays. ^a^log2 change represents fold difference of expression in wild type plants compared to *myb10myb72* mutants.
(DOCX)

Table S4 Primers used in this study.
(DOCX)
