# Supplementary figures and images for: MYB10 and MYB72 Are Required for Growth under Iron-Limiting Conditions
Source: PLoS Genet. 2013 Nov 21;9(11):e1003953. doi: 10.1371/journal.pgen.1003953 (PMC3836873; doi:10.1371/journal.pgen.1003953)

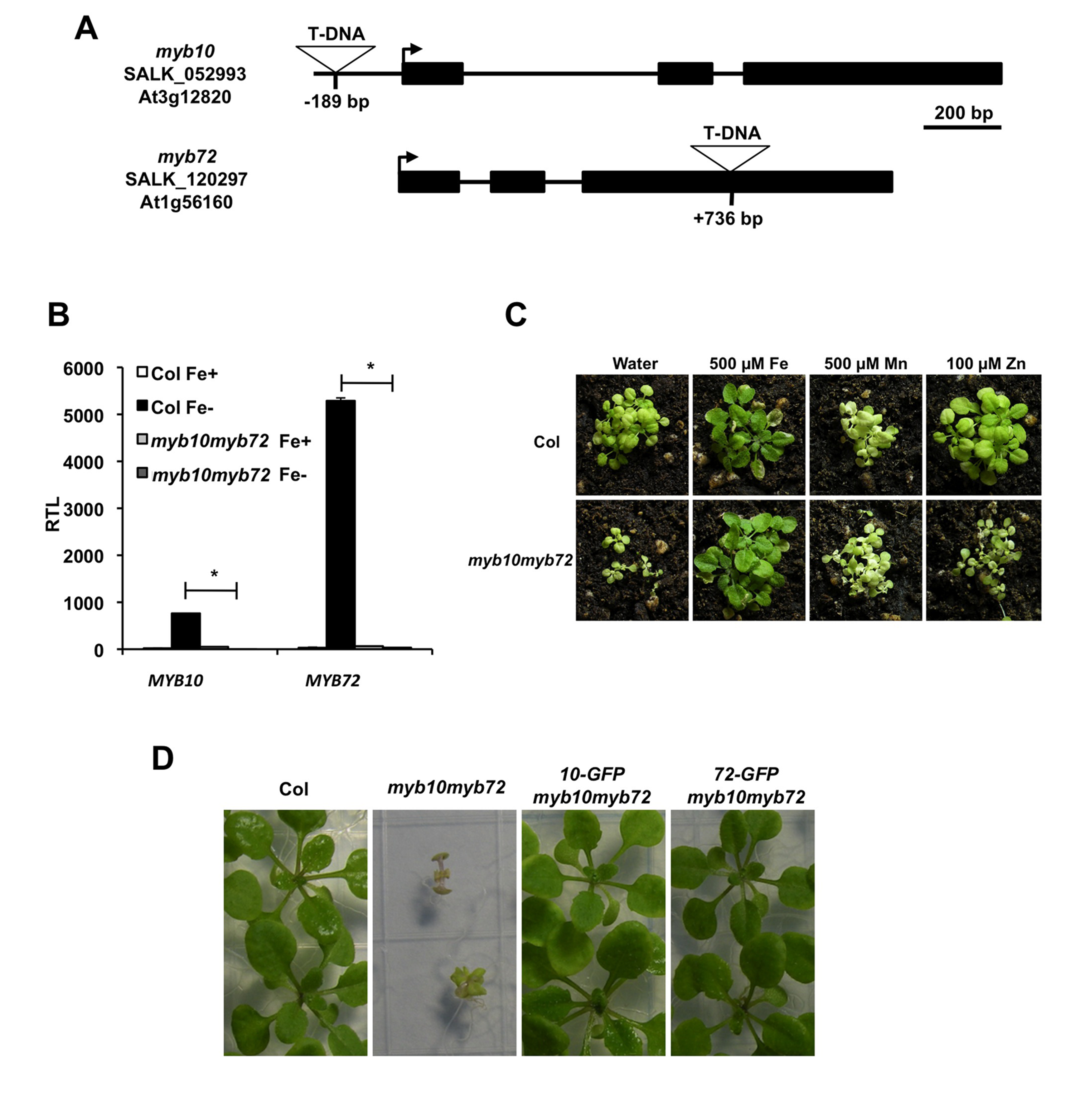

Supplement: Figure S1 — MYB10 and MYB72 are required under iron deficiency. A. Model of T-DNA insertion lines. Positions indicate distance from start codon. B. Relative transcript levels (RTL) as determined by qPCR on root tissue from plants grown for 2 weeks on ½ B5 and transferred to +/− Fe plates for 72 hrs. C. Plants were grown on alkaline soil (pH∼8) for 3 weeks and watered once a week with water, 500 µM FeEDDHA, 500 µM MnSO4, or 100 µM ZnSO4. D. Plants were grown for 3 weeks on ½ B5 plates with 50 µM NiCl2. *p<0.01. (TIF) [file pgen.1003953.s001.tif]

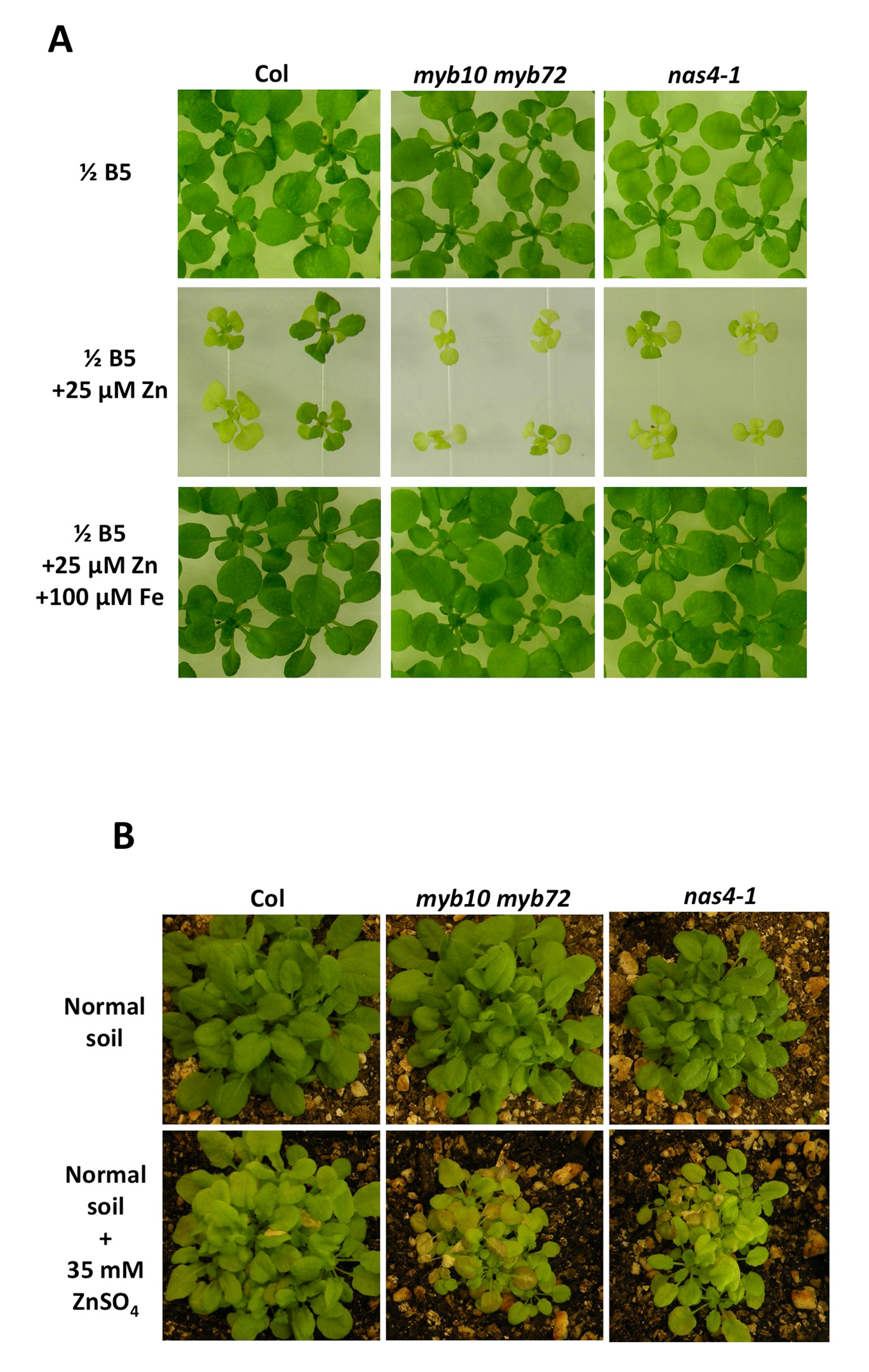

Supplement: Figure S2 — myb10myb72 and nas4-1 mutants are sensitive to excess zinc. A. Plants were grown for 3 weeks on ½ B5 plates supplemented as indicated above. B. Plants were grown on normal soil for 4 weeks and then soaked daily with 35 mM ZnSO4 for 11 days. (TIF) [file pgen.1003953.s002.tif]

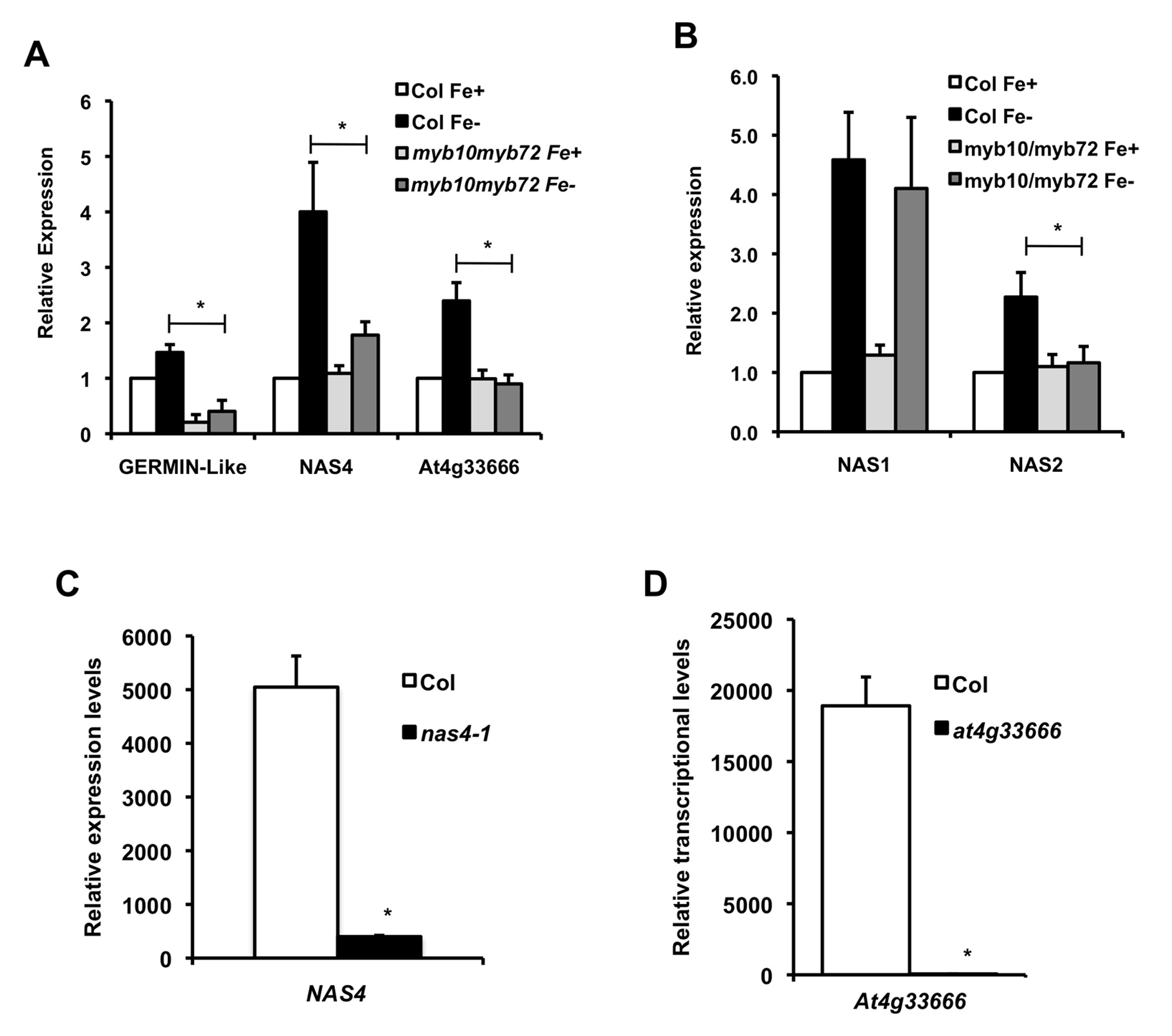

Supplement: Figure S3 — Identifying targets of MYB10 and MYB72. A., B. qPCR on root tissue from plants grown on ½ B5 for 2 weeks and transferred to +/−Fe for 72 hr. NAS3 is not expressed in the root. *p<0.05 C., D. qPCR analysis on roots of plants grown on ½ B5 for two weeks. (TIF) [file pgen.1003953.s003.tif]

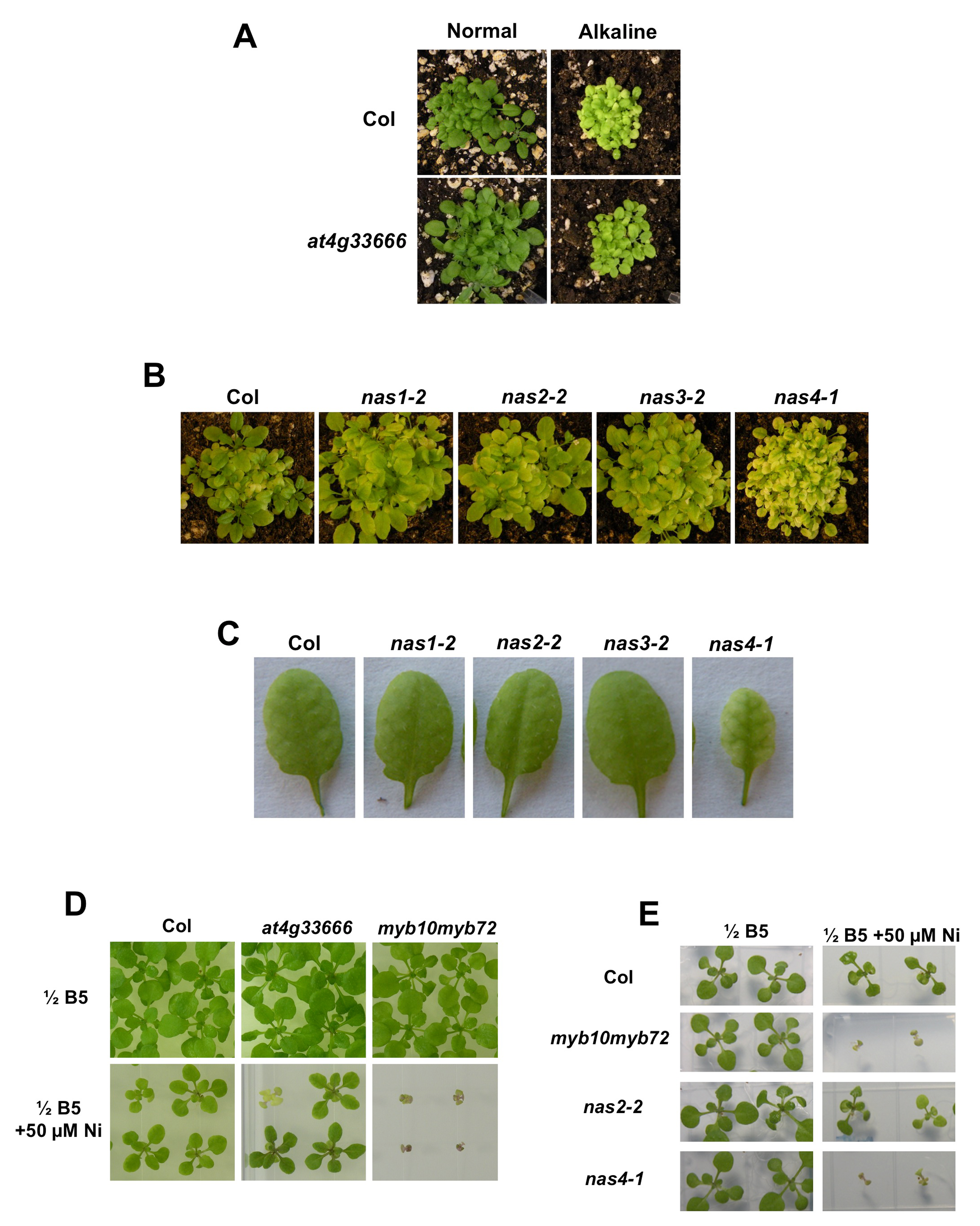

Supplement: Figure S4 — Analysis of other targets and NAS genes. A. Plants were grown on normal or alkaline soil for three weeks. B. Plants were grown for three weeks on alkaline soil. Homozygous mutants were tested from nas1-2 (SALK_082176), nas2-2 (SALK_066962), nas3-2 (SAIL_626_G10), and nas4-1 (SALK_130557). C. The fifth rosette leaf of plants grown as in B. D., E. Plants were grown on ½ B5 for 3 weeks and supplemented with 50 µM NiCl2 as indicated. (TIF) [file pgen.1003953.s004.tif]

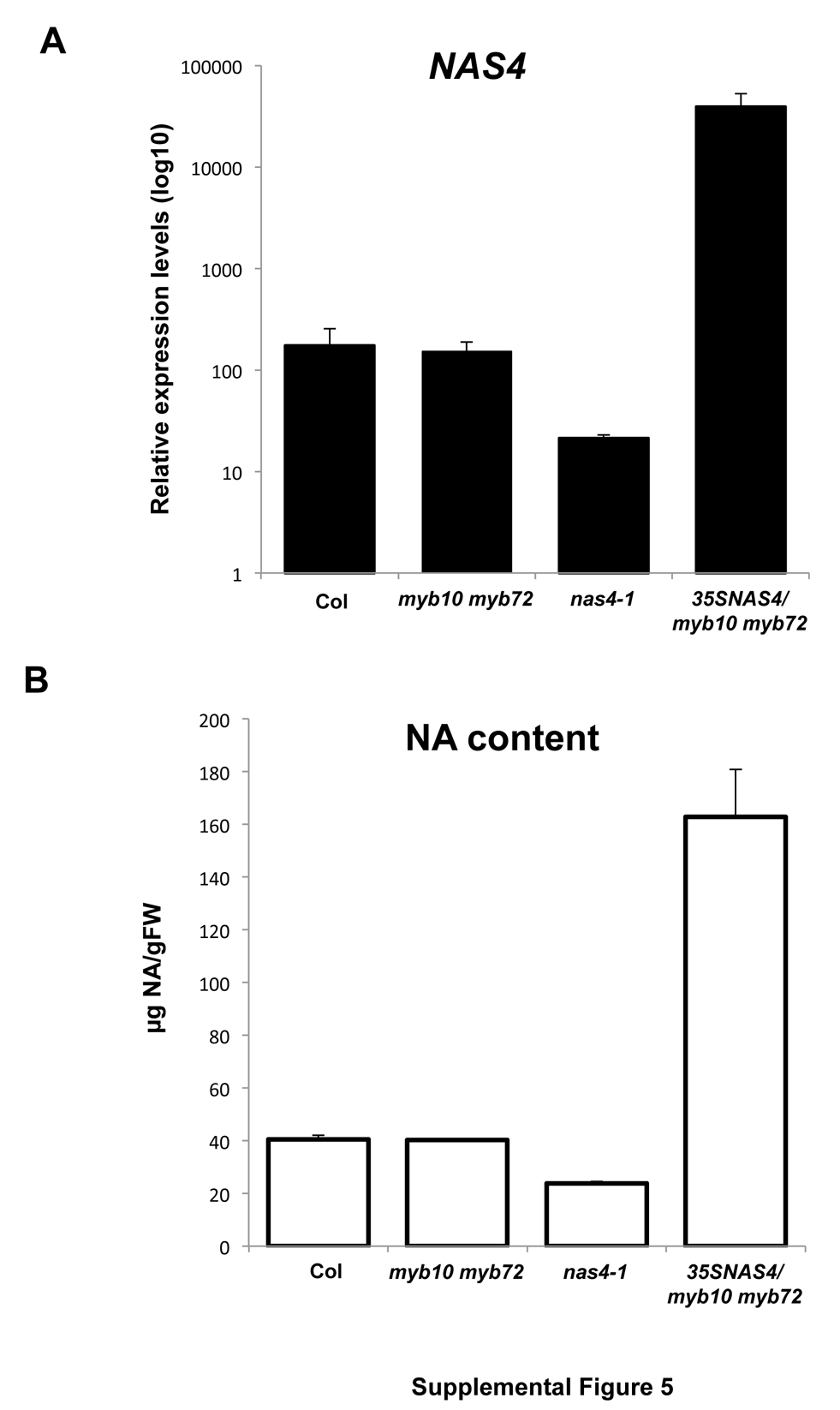

Supplement: Figure S5 — Overexpression of NAS4 increases NA content in myb10 myb72 plants. A. qPCR on plants grown for 2 weeks on 1/2 B5. B. NA content of same plants used in panel A. (TIF) [file pgen.1003953.s005.tif]

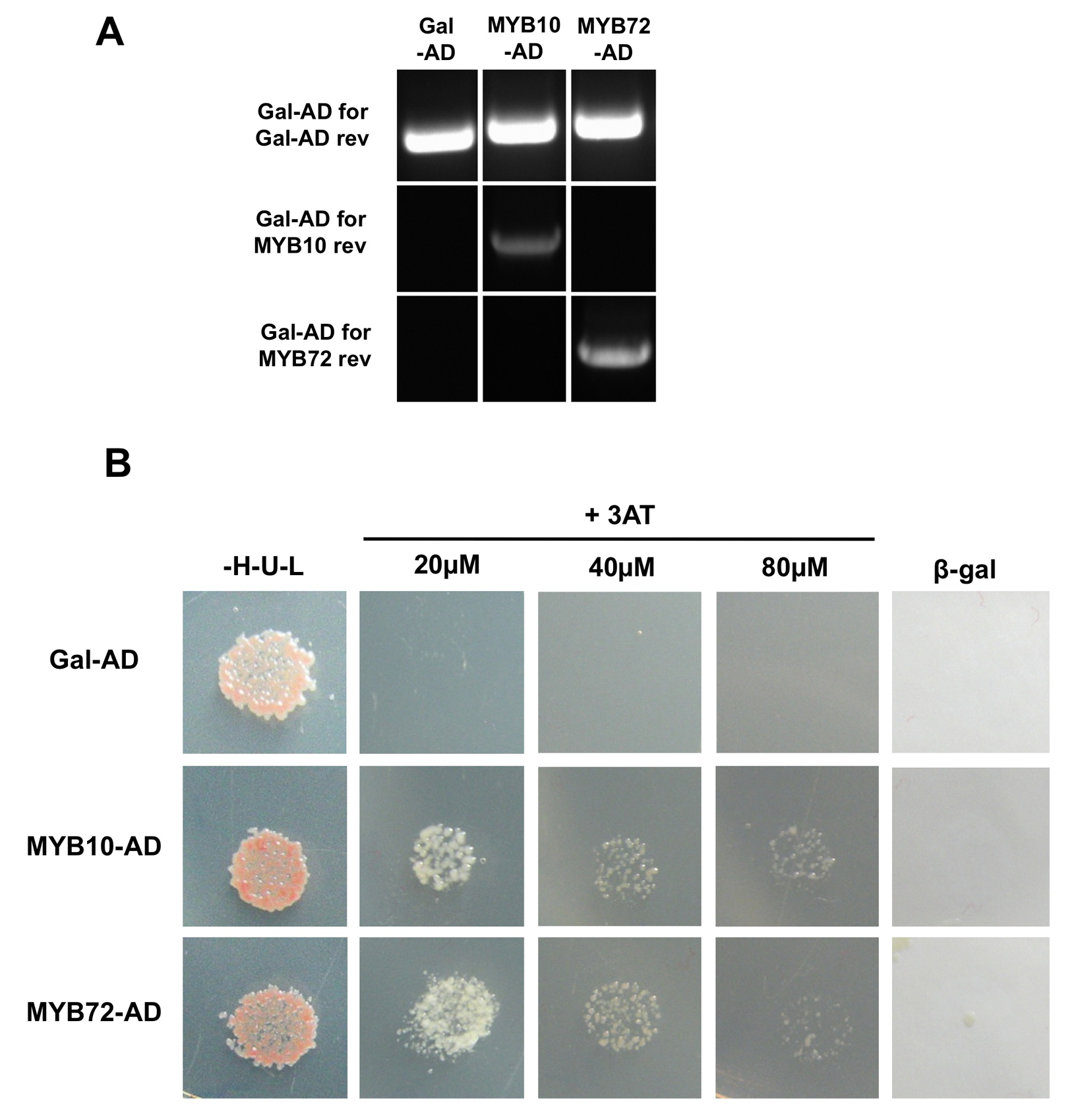

Supplement: Figure S6 — NAS2 and NAS4 are likely direct targets of MYB10/MYB72. A. Yeast stably expressing NAS4p-HIS3, NAS4p-LacZ were transformed with Gal-AD, MYB10AD, and MYB72-AD constructs. DNA was extracted and construct presence was confirmed by PCR. B. Colonies were grown on triple dropout selection plates and replica-plated onto media containing increasing concentrations of the HIS3 competitor 3AT and onto filter paper incubated with the β-gal substrate. C. ChIP analysis of the NAS2 and NAS4 promoter regions. (TIF) [file pgen.1003953.s006.tif]
