# Supplementary material for: MYB10 and MYB72 Are Required for Growth under Iron-Limiting Conditions
Source: PLoS Genet. 2013 Nov 21;9(11):e1003953. doi: 10.1371/journal.pgen.1003953 (PMC3836873; doi:10.1371/journal.pgen.1003953)
Supplement: Table S1 — ICP-MS data for hydroponically-grown roots. Metal levels in parts per million (ppm) were determined using ICP-MS analysis of tissue. Values represent mean ± SEM. n = 4 biological replicates. *significantly different from Col (p≤0.05). (DOCX) [file pgen.1003953.s007.docx]

**Table S1. ICP-MS data for hydroponically-grown roots**

|  | **Fe (ppm)** | **Cd (ppm)** | **Ni (ppm)** | **Zn (ppm)** | **Mn (ppm)** |
| --- | --- | --- | --- | --- | --- |
| **Fe+** | | | | | |
| Col | 463.6 ± 21.8 | 77.7 ± 17.9 | 6.3 ± 0.2 | 60.7 ± 3.8 | 5.6 ± 0.2 |
| *myb10myb72* | 445.8 ± 28.3 | 152.5 ± 42.3* | 6.8 ± 0.1* | 59.4 ± 4.2 | 5.9 ± 0.2 |
| *nas4-1* | 353.5 ± 58.3 | 80.5 ± 42.3 | 6.5 ± 0.1 | 51.0 ± 4.5 | 5.3 ± .01 |
| **Fe-** | | | | | |
| Col | 138.5 ± 18.5 | 283.6 ± 30.1 | 32.8 ± 3.5 | 113.5 ± 7.8 | 4.6 ± 0.1 |
| *myb10myb72* | 144.5 ± 20.5 | 282.4 ± 19.7 | 33.2 ± 4.2 | 104.2 ± 5.9 | 4.7 ± 0.2 |
| *nas4-1* | 165.7 ± 24.1 | 197.1 ± 24.3* | 26.9 ± 5.4 | 98.0 ± 8.5 | 4.6 ± 0.1 |

Metal levels in parts per million (ppm) were determined using ICP-MS analysis of tissue. Values represent mean ± SEM. n=4 biological replicates. *significantly different from Col (p ≤ 0.05).
