# Supplementary material for: MYB10 and MYB72 Are Required for Growth under Iron-Limiting Conditions
Source: PLoS Genet. 2013 Nov 21;9(11):e1003953. doi: 10.1371/journal.pgen.1003953 (PMC3836873; doi:10.1371/journal.pgen.1003953)
Supplement: Table S2 — ICP-MS data for soil-grown plants. Metal levels in parts per million (ppm) were determined using ICP-MS analysis of tissue. Values represent mean ± SEM. n = 4 biological replicates. *significantly different from Col (p≤0.05). (DOCX) [file pgen.1003953.s008.docx]

**Table S2. ICP-MS data for soil-grown plants**

|  | **Ni (ppm)** | **Mn (ppm)** |
| --- | --- | --- |
| **SHOOTS – normal soil** | | |
| Col | 1.4 ± 0.2 | 222.2 ± 4.4 |
| *myb10myb72* | 1.6 ± 0.1 | 188.1 ± 7.6* |
| *nas4-1* | 1.2 ± 0.1 | 193.5 ± 5.5* |
| **SHOOTS - intermediate soil** | | |
| Col | 1.3 ± 0.2 | 75.6 ± 1.1 |
| *myb10myb72* | 1.5 ± 0.1 | 42.2 ± 1.1* |
| *nas4-1* | 1.0 ± 0.1 | 69.9 ± 1.5* |
| **SEEDS – normal soil** | | |
| Col | 0.15 ± 0.02 | 47.6 ± 1.1 |
| *myb10myb72* | 0.07 ± 0.01* | 52.0 ± 1.8 |
| *nas4-1* | 0.10 ± 0.02* | 46.3 ± 1.2 |

Metal levels in parts per million (ppm) were determined using ICP-MS analysis of tissue. Values represent mean ± SEM. n=4 biological replicates. *significantly different from Col (p ≤ 0.05).
