# Supplementary material for: MYB10 and MYB72 Are Required for Growth under Iron-Limiting Conditions
Source: PLoS Genet. 2013 Nov 21;9(11):e1003953. doi: 10.1371/journal.pgen.1003953 (PMC3836873; doi:10.1371/journal.pgen.1003953)
Supplement: Table S3 — 1.5-fold misregulated genes by microarray. Plants were grown for 2 wk on ½ B5+2% sucrose and transferred to −Fe conditions for 72 hr. RNA was prepared from root tissue and hybridized to GeneChip Affymetrix Arabidopsis ATH1 Genome Arrays. alog2 change represents fold difference of expression in wild type plants compared to myb10myb72 mutants. (DOCX) [file pgen.1003953.s009.docx]

**Table S3. 1.5-fold misregulated genes by microarray**

| **Probe** | **AGI** | **NAME** | **Predicted function** | **log2 change^a^** |
| --- | --- | --- | --- | --- |
| 257689_at | AT3G12820 | MYB10 | MYB txn factor | 4.07 |
| 262091_at | AT1G56160 | MYB72 | MYB txn factor | 3.38 |
| 249476_at | AT5G38910 | germin-like protein | putative MnSOD | 2.72 |
| 253305_at | AT4G33666 | unknown protein | unknown protein | 1.98 |
| 259632_at | AT1G56430 | NAS4 | nicotianamine synthase | 1.09 |
| 249636_at | AT5G36890 | glycosyl hydrolase family 1 | glycosyl hydrolase family | 1.08 |
| 260551_at | AT2G43510 | ATTI1 (DEFL family) | Putative trypsin inhibitor protein-herbivory defense | 1.07 |
| 245319_at | AT4G16146 | unknown protein | similar to negatively light-regulated protein | 1.01 |
| 248585_at | AT5G49640 | unknown protein | unknown | 1.04 |
| 264710_at | AT1G09790 | COBL6 |  | 0.997 |

Plants were grown for 2 wk on ½ B5 + 2% sucrose and transferred to –Fe conditions for 72 hr. RNA was prepared from root tissue and hybridized to GeneChip Affymetrix Arabidopsis ATH1 Genome Arrays.

^a^log2 change represents fold difference of expression in wild type plants compared to *myb10myb72* mutants.
