# Supplementary material for: MYB10 and MYB72 Are Required for Growth under Iron-Limiting Conditions
Source: PLoS Genet. 2013 Nov 21;9(11):e1003953. doi: 10.1371/journal.pgen.1003953 (PMC3836873; doi:10.1371/journal.pgen.1003953)
Supplement: Table S4 — Primers used in this study. (DOCX) [file pgen.1003953.s010.docx]

**Table S4. Primers used in this study**

| **Primer** | **Sequence** |
| --- | --- |
| NAS1 qPCR FP | GCTCTGAGAGCATGAGCACTCCTT |
| NAS1 qPCR RP | GGCCTTGACCAATATGACGTCGTT |
| NAS2 qPCR FP | GGTCTAACCCCTCCTTAGCGTT |
| NAS2 qPCR RP | CCGGTCCGATGCCACTTACTTCCAT |
| NAS4 qPCR FP | CTGTGGTGAGGCTGAAGGTTACT |
| NAS4 qPCR RP | GAGGGAGAGGACCAGAGCCAA |
| EF-1α qPCR FP | CAGTCATTGATGCCCCAGGAC |
| EF-1α qPCR RP | TGTTGTCTCCCTCGAATCCAGAG |
| MYB72 qPCR FP | GACTCGAGAGGTAACCAAATCG |
| MYB72 qPCR RP | GTTGAACCACTCGTCGTACTC |
| MYB10 qPCR FP | CGTTTGAGACCTGGTCTCAGATAG |
| MYB10 qPCR RP | GCCATGGAGTGATGAAGAAAGTG |
| At4g33666 qPCR FP | GGCCTCATCAGCTGCGATG |
| At4g33666 qPCR RP | CGGTTCCGGTGTGGCTGTA |
| MYB10 start FOR | CACCATGGGGAATAGAAGAGCACC |
| MYB10 nostop REV | GCAAGTTTGGTTATCCAAGAAAATGAGC |
| MYB10 stop REV | CTAGCAAGTTTGGTTATCCAAGAAAA |
| MYB72 start FOR | CACCATGGGGAAAGGAAGAGCACC |
| MYB72 nostop REV | TAGACATACTTCTCCGACGAAATT |
| MYB72 stop REV | TCATAGACATACTTCTCCGACG |
| MYB10 -1.5kb FOR | CACCGTAGAAAAATGCAGGAATCC |
| MYB10 -1 bp REV | CACACCCCACTTTTAATTTTAGAGAATC |
| MYB72 -1.5kb FOR | CACCGCAATCTTACATCAGAGCCTTGTGG |
| MYB72 -1 REV | TCTTATTACACTACTTTCTTCTCTATAGCTACC |
| NAS4 start FOR | CACCATGGGTTATTGCCAAGACG |
| NAS4 stop REV | CTAGGTAAGTTGTTCTTCATTAGCACC |
| NAS4 -1.5kb FOR | CACCGATTGGAGAGTTGTAGCTTTATGC |
| NAS -1 bp REV | GTCGACACTATGAGGTTTTACGAG |
